# Assessment of the Phytochemical Analysis and Antimicrobial Potentials of *Zingiber zerumbet*

**DOI:** 10.3390/molecules28010409

**Published:** 2023-01-03

**Authors:** Muhammad Ramzan, Basit Zeshan

**Affiliations:** 1 Department of Microbiology, Faculty of Science and Technology, University of Central Punjab, Lahore 54782, Pakistan; 2 Faculty of Sustainable Agriculture, University Malaysia Sabah, Sandakan Campus, Locked Bag No. 3, Sandakan 90509, Sabah, Malaysia

**Keywords:** *Z. zerumbet*, medicinal plants, plant extracts, antimicrobial resistance, chemical composition, HPLC

## Abstract

Antimicrobial resistance (AMR) has arisen as a global concern in recent decades. Plant extracts used in combination with antibiotics are promising against AMR, synergistically. The purpose of this study was to evaluate the component of the bitter ginger (*Zingiber zerumbet*) extract in different solvents using high-performance liquid chromatography (HPLC), in addition to evaluate the antibacterial activity of these extracts, in combination with their antibiotic potential against four multi-drug resistant (MDR) bacterial strains (*Lactobacillus acidophilus*, *Streptococcus mutans*, *Enterococcus faecalis* and *Staphylococcus aureus)*. Ethanol and the aqueous extracts of bitter ginger were prepared using a conventional solvent extraction method and were evaluated for their phytochemistry using HPLC, qualitatively and quantitatively. Moreover, the antibiotic susceptibility of the pathogenic isolates was determined. A disc diffusion assay was used to obtain the antimicrobial potential of the extracts alone and with antibiotics. Eight components were identified from the separation of the bitter ginger extract by HPLC. For AMR bacteria, the combination of the antibiotic solution with the bitter ginger crude extracts could improve its susceptibility of these antibiotics. This study indicates that the combination of an antibiotic solution with the bitter ginger crude extract exhibits potent antibacterial activities against MDR bacterial strains. Therefore, they can be used for the treatment of various diseases against the microbial pathogen and can be incorporated into medication for antibacterial therapy.

## 1. Introduction

The scientific name of bitter ginger is *Zingiber zerumbet* (L.) Roscoe ex Sm. [1]. It belongs to the family *Zingiberaceae,* the largest family of the order Zingiberales with about 57 genera of 1600 species worldwide [2,3]. The eminent members of this family, such as *Curcuma longa* (turmeric) [4], *Zingiber officinale* (ginger) [5], *Elettaria cardamomum* (cardamom) [6], and *Zingiber zerumbet* (bitter ginger), are used in folk medicine, agriculture, food condiments, and for ornamental purposes [7]. Among the member *Zingiber zerumbet* is commonly known as bitter ginger or shampoo ginger and is widely grown in both tropical and subtropical areas of the world [8]. Occasionally, the rhizome is the most widely explored part of the plant for its aromatic compounds. In addition, the rhizome is also reported to be rich in terpenoid (mono-terpenoids, sesquiterpenoids, and steroids) phenolic (tannins), alkaloids aromatic compounds, vanilline, zerumbone, phenolic, and flavonoid, especially kaemferol and its glucoside, along with curcumin and ginerol [9,10,11]. Zerumbone is well-known for its antimicrobial, a powerful antioxidant agent, anti-inflammatory, anticancer properties, and chondro-protective properties [12]. Other compounds of *Zingiber zerumbet,* such as zederone, have also reported antimicrobial activities against MDR bacterial strains, such as *Staphylococcus aureus*, Bacillus spices, and *Pseudomonas aeruginosa*. Extracts of bitter ginger (rhizome) were previously used to cure several diseases and other pathological conditions, including pain, arthritis, microbial or viral infections, gastroenteritis, anti-ageing, diabetes, gout, gastric ulcers, anti-inflammatory and anti-allergic conditions, skin ailments, and cancer [13].

Bitter ginger and its extracts have been used as a treatment for various infections for many years for their antimicrobial properties [12,14]. It shows an antimicrobial activity against many Gram-negative bacteria, such as *Escherichia coli*, *Helicobacter pylori,* and Gram-positive bacteria, such as *Staphylococcus aureus*, *Staphylococcus epidermidis,* showing a greater antibacterial effectiveness on Gram-positive organisms [12,14]. Additionally, more studies are need to determine the potential of bitter ginger as an antibacterial agent against Gram-positive microorganisms, such as *Streptococcus mutans*, the main agent of tooth decay, the oral infection disease most prevalent in the world that affects over 90% of school-aged children and about 100% of the world population [15].

Ethanol is a suitable solvent for the active substance in *Zingiber zerumbet* in the form of alkaloids, tannins, flavonoids, and terpenoids [16], and is a versatile solvent that has the ability to extract with a broad polarity, ranging from non-polar compounds to polar compounds [17]. Water (distilled water) is also considered as environmentally friendly and is generally recognized as a safe solvent by food, and nutraceutical industries. It has many advantages and plays a role as a low cost “green solvent” because it is natural, is widely available, has a high purity, and is non-toxic. The use of distilled water and an ethanol solvent for the extraction process of the *Zingiber zerumbet* extract will result in the profile difference of the active compound of the extract, which then will affect the biological activities of the extract.

The increasing antimicrobial resistance (AMR) among common pathogens has raised major concerns among physicians and medical professionals [18,19,20]. With the increase in AMR and the emergence of MDR organisms, such as *Klebsiella pneumonia*, *Escherichia coli*, *Pseudomonas aeruginosa*, *Staphylococcus aureus*, and *Enterococcus faecalis,* treatment options have been reduced [21,22,23]. These pathogens of the oral cavity, skin, intestinal tract, etc., are of great public health concern. The unavailability of new drugs, the misuse of antibiotics, and the emergence of drug-resistant organisms, are leading causes of death worldwide [24,25]. People are now moving towards herbal medicines and traditional remedies to overcome antibacterial resistances against available antibiotics [11].

The current study was designed to evaluate and highlight the importance of the commonly available medicinal plants, such as bitter ginger, as a treatment option. In this study, different components of bitter ginger (*Z. zerumbet*) were extracted by aqueous and ethanol solvents and analysed for a phytochemical analysis, and the antibacterial activity against selected MDR pathogens was evaluated.

## 2. Results

### 2.1. Phytochemical Analysis

The phytochemical analysis of the extracts (aqueous and ethanol) of bitter ginger (*Z. zerumbet*) revealed the presence of a significant number of secondary metabolites. Table 1 lists the tests used and the different solvent extracts of bitter ginger (*Z. zerumbet*), assessed qualitatively, through a phytochemical analysis, respectively.

### 2.2. HPLC Analysis

The HPLC analysis of the crude extract of *Z. zerumbet* (aqueous extract), allowed for the identification of eight compounds, in comparison with the standard. Comparing the phenolic and flavanol retention times to the reference standards, allowed for the identification (Sigma Chemicals Co., St Louis, MO, USA). The RT for each analyte was used to pick a similar peak (in terms of the retention time), as that of the standards. The HPLC program has the ability to detect the flavonoid and phenolic compounds that showed similar peaks with similar retention time as that of the standards. This provided a base for the identification of each analyte.

**Figure 1:**
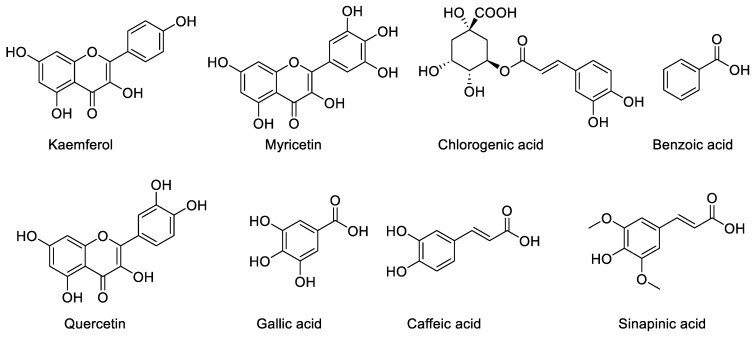
(**A**) The chromatogram of the standard phenolic compounds. (**B**) The chromatogram of the standard flavonoid compounds.

**Figure 2:**
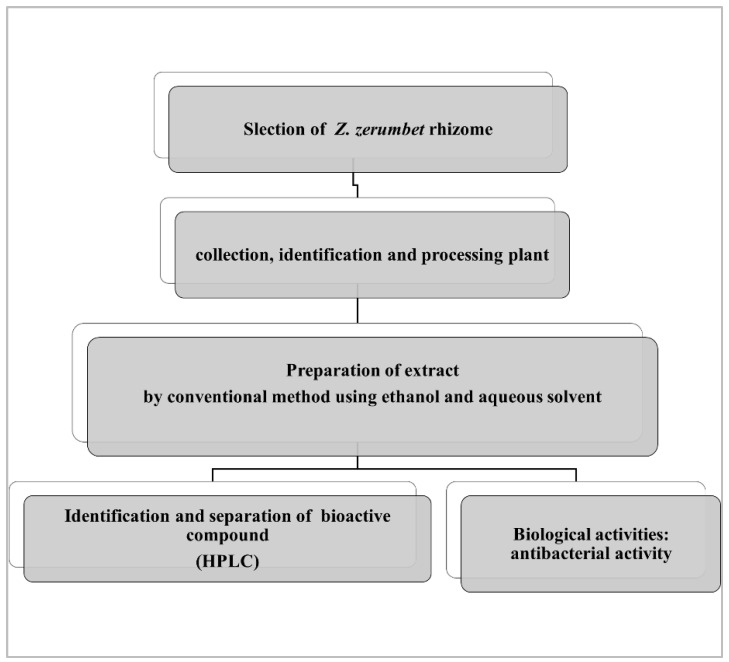
The chemical structure of the phenolic compounds.

The identified components of the *Z. zerumbet* extract are caffeic acid, kaempferol, gallic acid, quercetin, sinapic acid, chlorogenic acid, benzoic acid, and myricetin. The identified components from the class of phenolic compounds are chlorogenic acid (16.92%, 16.67%), gallic acid (0.723%, 9.48%), sinapic acid (0.50%, 3.13%), caffeic acid (2.36%. 1.86%), myricetin (26.80%, 39.34%), benzoic acid (2.57%, 0%), kaempferol (0.06%, 0.45%), and quercetin (0.31%, 0.78%) in the aqueous and ethanolic extract, respectively. There are differences in the percentage of these components in the two crude extracts, according to the % peak area, which is illustrated in Table 2. The HPLC analysis of the bitter ginger extract is present in Figure 1 and Figure 2). Other peaks in the HPLC analysis show other biomolecules, which are present in the *Z. zerumbet* extract.

Benzoic acid was the compound and it is one of the phenolic compounds present in the aqueous extract of *Z. zerumbet,* but it is not present in the ethanolic extracts.

The HPLC analysis of the standard phenolics and flavonoids is given in Figure 1 and Table 3. HPLC analysis of the various extracts (organic/aqueous) of *Z. zerumbet*, the peak assignment of the component includes chlorogenic acid, gallic acid, sinapic acid, caffeic acid, myricetin, benzoic acid, kaempferol, and quercetin.

### 2.3. Antibacterial Activity of the Plant Extract Combined with the Antibiotic Solution

When the antibiotics were tested without the prepared extracts, the tested bacterial strains showed resistance against the tested drugs. *Lactobacillus acidophilus* showed a resistance against ciprofloxacin, chloramphenicol, and tetracycline. *Streptococcus mutans*, *Enterococcus faecalis,* and *Staphylococcus aureus* showed resistance to all of the tested drugs, to varying degrees, as shown in Table 4.

The synergistic mixture used in the study of the antibiotics and bitter ginger (aqueous and ethanolic) extracts is a combination of antibiotics at a concentration of 30 µg /mL (except CIP 5 µg/ mL) and the bitter ginger (aqueous and ethanolic) extracts at a concentration 10 mg/mL. When the extracts combined with antibiotics were used, the different resistance patterns of the tested organisms were observed, as shown in Table 4 and Table 5. The larger ZOI was observed and noted for the antibiotics treated with the bitter ginger aqueous and ethanolic extracts.

## 3. Materials and Methods

### 3.1. Extraction

**Figure 3:**
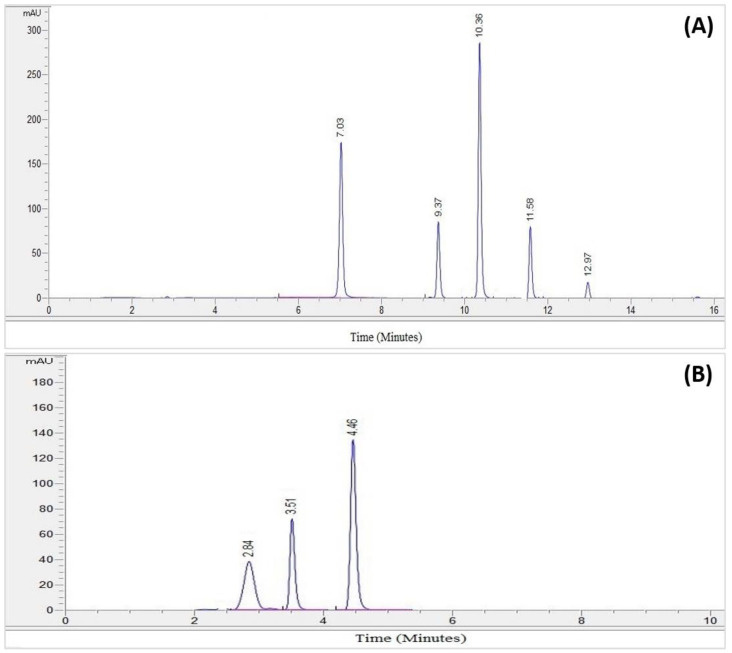
General flow chart of the plant extraction and biological activity.

A fresh rhizome of a *Z. zerumbet* sample was obtained from the local market of Old Lahore (the walled city of Lahore). Following the collection, the rhizomes were washed thoroughly with distilled water, shade dried (one month), and then identified using their vernacular name by the Department of Botany, University of the Punjab, Lahore (voucher Number: LAH#280921). Following the shade drying step, the grinding of the plant was carried out to convert it into a fine powder, approximately 500 g, and the powder was processed for aqueous extraction. The aqueous (distilled water) and 95% ethanol extracts of the bitter ginger rhizome were prepared. Samples of 50 g were extracted for 5 days with 250 mL of the respective solvent, at 40 °C, in a shaking incubator at 200 rpm. Then, the extracts (mixtures) were initially filtered twice with nylon cloth, to remove the solid residue and then were further filtered using a Whatman filter paper. The fresh filtrate was left to evaporate using a rotary evaporator [26,27]. The final weighted extract was transferred to a clean airtight glass bottle and stored at 2 to 8 °C, and processed for the phytochemical analysis, and antibacterial activities (Figure 3).

### 3.2. Preliminary Phytochemical Screening

A preliminary phytochemical examination was performed using the standard protocols, to test for the existence of glycosides, tannins, alkaloids, flavonoids, terpenoids, and quinones, qualitatively [28]. To determine the presence of alkaloids, Wagner’s test was used. To check for saponins, in six test tubes, 5 mg of the powdered extract was added, followed by 20 mL of 95% ethanol. The mixture was subjected to stirring for 12–15 min. The soapy appearance of the sample indicated the presence of saponin. To test for the presence of tannins, six tubes were loaded with 5 mg of the powdered extract and 5 mL of 95% ethanol, followed by the addition of 5% ferric chloride. The appearance of blue or a dark colour was indicative of the presence of tannins. Then, 3 mL of sodium hydroxide solution was added to each tube containing flavonoids extracted with six different solvents. A gentle stirring produced the colour, and its absence showed flavonoids. For the terpenoids test, 5 mg of the extracts were dissolved in 2 mL of pure chloroform and 5M H_2_SO_4_ was added to it. A red-brown precipitate suggested terpenoids [29]. To test for glycosides, 5 mg of powdered extracts were placed in six tubes (separately) with 5 mL of six different solvents, 2 mL of glacial acetic acid, 2% ferric chloride, and 1 mL of H_2_SO_4_. At the interface of the two liquids, glycosides produce a brown or violet ring [30]. Quinone was extracted six times with concentrated H_2_SO_4_. Red precipitates indicated the presence of quinones [31].

### 3.3. High-Performance Liquid Chromatography Analysis

The *Z. zerumbet* extracts were analysed for the phytochemical analysis using high-performance liquid chromatography (HPLC) techniques. Eight standards were tested, i.e., chlorogenic acid, quercetin, sinapic acid, gallic acid, benzoic acid, kaempferol, caffeic acid, and myricetin using HPLC. For this, 5 mg of the powdered materials of the standards and plant extract were dissolved in 2 mL of 95% ethanol, followed by filtration (0.45 µm syringe filter). An HPLC instrument (model 1260 Agilent, CA, USA) equipped with a quaternary pump 1260 and a DAD detector was used. For the data analysis, ChemStation software was used. A 20 µL volume of the filtered sample was injected into Zorbax Eclips Plus (Agilent, CA, USA), using a reverse phase (C18) column (4.6 × 250 mm; 5 µm particle size). The mobile phase used was a mixture of distilled methanol (solvent A) and 1% acetic acid (solvent B). The flow rate was of 1 mL/min in a linear gradient, following the scheme (t in min; %A): (0; 60%), (5; 35%), (10; 10%), (15, 60%), and (20; 60%). The column temperature was maintained at 25 °C. The chromatograms were recorded at 280 nm. In the case of the flavonoids, two solvent systems were utilized for the mobile phase: A, which included 3% trifluoroacetic acid, and B, which contained acetonitrile and methanol (80:20 *v*/*v*), respectively. The mobile phase, a mixture of solvents A and B (50:50 *v*/*v*), which was first filtered under vacuum through a 0.45 µm membrane, was eluted by isocratic elution at a flow rate of 1 mL/min at 30 °C. A wavelength of 360 nm was used for the detection process.

### 3.4. Collection and Preparation of the Bacterial Strains

For testing the antibacterial activity, the *Lactobacillus acidophilus*, *Streptococcus mutans*, *Enterococcus faecalis,* and *Staphylococcus aureus* strains were obtained from the laboratory of Microbiology, the University of Veterinary and Animal Sciences (UVAS), Lahore, Punjab, Pakistan. In the UVAS, these bacterial strains were isolated and identified from different animal and human clinical samples, and then saved for further research purposes in the bacterial strains store bank. First, these bacterial strains were sub-cultured on blood agar, nutrient agar, and nutrient broth, to obtain the fresh bacterial colonies. The plates were incubated at 37 °C for 24 h. Following the incubation period, the bacterial colonies were identified using different biochemical tests, including catalase, coagulase, DNA, and the bile esculin test [24,32,33].

#### 3.4.1. Antibiotic Susceptibility of the Four Pathogenic Strains

The disk diffusion method was used, as per the standard guidelines from the Clinical Laboratory Standard Institute (CLSI) [32]. First, the antibacterial patterns of the bacterial isolates were obtained without any bitter ginger extract, using the disk diffusion technique. For this, 0.5% McFarland standard was prepared using Turbidimeter (Oxoid, Basingstoke, UK) for each bacterial isolate. This suspension was spread onto a Muller Hinton (MH) agar to make a bacterial lawn. Antibiotics (discs) of tetracycline (30 µg), chloramphenicol (30 µg), and ciprofloxacin (5 µg) were placed onto the agar plates followed by an incubation period of 24 h at 37 °C. Following the incubation period, the zone of inhibition (ZOI) was measured and noted.

#### 3.4.2. Preparation of the Synergistic Mixture for the Antibacterial Activity

The synergistic mixture was prepared in different types of combination of antibiotics and plant extracts. It was prepared with a combination of plant extracts (aqueous and ethanolic) at a concentration of 10 mg/mL and different antibiotics at a concentration of 30 µg/mL, respectively, in 1:1 ratio, except for ciprofloxacin (concentration 5 µg/mL)

#### 3.4.3. Antibacterial Effect of the *Z. zerumbet* Extracts Combined with Antibiotics

The synergistic effect of the bitter ginger extracts (aqueous and ethanolic) and three common antibiotics was investigated. The antibiotics discs were tetracycline (TE, 30 µg), chloramphenicol (C, 30 µg), and ciprofloxacin (CIP, 5 µg). As a control, the antibiotics were tested without the bitter ginger extract (2.5.1). For studying the synergistic effect of the bitter ginger extracts with the antibiotic solution. 20 µL of the desired mixture was added to the paper disc. A disc of each set was displaced on an agar plate inoculated with the tested bacterium. The zones of inhibition produced by the plant extract with the antibiotic’s solution after the incubation period at 37 °C for 24 h, were estimated. One percent di-methyl sulfoxide (DMSO) was used as a negative control for the test. If the zone of the combination treatment was greater than the zone of the plant extract plus the zone of the corresponding antibiotics, this was interpreted as synergism; if it was equal to the zone of the plant extract plus the zone of the corresponding antibiotics, this was interpreted as additive; and if it was less than the zone of the plant extract plus the zone of the corresponding antibiotics, this was interpreted as antagonism. The activity was described as sensitive and resistant by a zone of inhibition (ZOI). A zone diameter of ≥21 mm was deemed sensitive for CIP, C. The sensitivity zone for TE was around 23 mm. The Clinical and Laboratory Standard Institute’s guidelines (2017) were used to see the zone of inhibition for the antibacterial test [34].

### 3.5. Statistical Analysis

The data were analysed using the Statistical Package for Social Sciences (SPSS version 20). The standard deviation and mean values were used to define the data. The ZOI was also calculated in terms of the standard deviation.

## 4. Discussion

The present study was designed to obtain the preliminary information on the in vitro antimicrobial activities of the bitter ginger extract combined with an antibiotic solution on four MDR bacterial strains. A disc diffusion method was preferred to be used in this study, two crude extracts were analysed using HPLC. The results showed that the extraction using distilled water and ethanol has antimicrobial activity against the bacterial strains. This may be because of the presence of phenolic, flavonoid, and other bioactive compounds in the bitter ginger extract [35]. Moreover, our results showed that there is a noticeable difference in the antimicrobial activity of the extract of bitter ginger when distilled water is the solvent, than the extract when ethanol is the solvent, as seen in Table 4 and Table 5. There are two points which can help explain this. The first point is that, as a result of the variation in the chemical composition between the two bitter ginger extracts, i.e., the variations related to the presence of benzoic acid in the aqueous extract but not in the ethanolic extract, as determined in Table 2. The second point is that because of the difference in the concentrations of materials, i.e., benzoic acid in the two extracts, this may lead to a different antimicrobial potency, this difference in concentration could be due to the nature of the solvent used for the extraction. Furthermore, the results for both extracts, combined with the antibiotic solution, were effective against all strains. This is due to the negative charge of the surface of the Gram-positive wall, which may reduce their resistance to an antibacterial component.

The phenolic acid of the extract of bitter ginger was detected using HPLC. The seven standards available are: chlorogenic acid, quercetin, sinapic acid, gallic acid, benzioc acid, kaempferol, caffeic acid, and myricetin [7]. In the current study we found various phenolic and flavonoid compounds in bitter ginger, including chlorogenic acid, quercetin, sinapic acid, gallic acid, benzioc acid, kaempferol, caffeic acid and myricetin. The current study demonstrated that the amount of phenolic and flavonoid compounds differed greatly from one extract to the next. These phenolic acids are found naturally in medicinal plants, reported to be prominent bioactive compounds in most medicinal plants. The findings in this study agreed with a previous study [2]. Elguindy et al., 2016 found that the main phenolic compound identified in the cardamom extract (*Zingiberaceae*), was caffeic acid. The aqueous extracts showed the presence of the highest concentrations of chlorogenic acid. Similar findings were reported in previous studies, in which they reported that compounds in different extracts differed in concentrations [36].

In the current study, generally, *Z. zerumbet* extracts (aqueous and ethanol) did not show any antimicrobial potential against all strains. Therefore, there were no synergistic effects between the *Z. zerumbet* extracts (aqueous and ethanol) and the three different antibiotics studied against the resistant bacterial isolates. However, there was considerable improvement in the ciprofloxacin, chloramphenicol, and tetracycline action when the *Z. zerumbet* extracts were added to the ciprofloxacin, chloramphenicol, and tetracycline solutions (Table 4 and Table 5). This interesting finding was in harmony with those demonstrated by [37]. They stated that, the antibacterial activities of some antibiotics, including ampicillin, kanamycin, erythromycin, and chloramphenicol, have been enhanced in the presence silver nanoparticles against Gram positive and Gram-negative bacteria. Generally, the obtained result demonstrated that the combination of antibiotics with the *Z. zerumbet* extracts have a better antimicrobial potential. Bitter ginger’s antimicrobial properties were the primary rationale for its use, implying that ginger itself or its extracts could be used as a treatment for infections of a bacterial origin [6]. Combining two drugs may have additive, antagonistic, or synergistic effects. Many in vitro studies have shown that combining plant extracts with antibiotics has a synergistic impact that lowers the level of MICs for the antibiotics significantly. The combined action of an antibiotic drug and a plant extract against certain MDR bacterial strains was one of the primary goals of the current investigation. The results of this research may aid in understanding the synergistic impact of combination therapy, which, in more recent times may have the potential to provide a fresh approach to the management of bacterial infections. Antibiotic plant extracts showed a synergistic efficacy between the plant extracts and antibiotics against the MDR bacterial isolates shown to be effective previously. At current, tetracycline, ciprofloxacin, and chloramphenicol were used. According to the findings of this study, *Lactobacillus acidophilus*. *S. mutans,* and *E. faecalis Staphylococcus aureus* were found to be the most susceptible to ethanol extracts containing antibiotics, as compared to the aqueous extract containing antibiotics.

*Staphylococcus* spp. is resistant to the anti-biofilm effects of quercetin, which impairs the quorum sensing. Additionally, quercetin is a well-known cancer-fighting and anti-oxidant agent [38,39]. In the current study, quercetin was abundantly detected in the *Z. zerumbet* extract, but due to their high polarity, their concentrations were highest in the ethanol and water extracts of *Z. zerumbet*. Saponins, tannins, glycosides, terpenoids, quinones, and flavonoids were found in the present study. Saponins have the ability to deal with bacteria and fungi [40]. Flavonoids are the main substance having significant anti-oxidation and antimicrobial potentials [41].

## 5. Conclusions

The results of the present study have shown that the *Z. zerumbet* extracts have sufficient antibacterial activities for their various solvent-based extracts. The combination of the plant extract with antibiotics will open new approaches in the pharmaceutical sector for the manufacture of pharmaceutical products, which may help in utilizing these extracts in the treatment of various infections.

## Figures and Tables

**Table 1:** Phytochemical analysis of the extracts of bitter ginger (*Z. zerumbet*).

Phytochemical Analysis	Ethanol	Aqueous
Tannins	−	+
Terpenoids	+	+
Saponin	+	+
Cardiac glycoside	+	+
Quinones	−	−
Flavonoids	+	−
Alkaloids	+	+

+: Positive. −: Negative.

**Table 2:** Flavonoid and phenolic contents in the *Z. zerumbet* rhizome extract, determined by the HPLC methods.

Plant Name	Phenolic and Flavonoid Compounds	Retention Time	Area%	Wavelength nm	mg/g
ZZAE	Benzoic acid	12.818	2.5772	280	7.289 ± 0.85
	Gallic acid	7.02	0.7234		0.265 ± 0.41
	Sinapic acid	11.662	0.5014		0.22 ± 0.03
	Caffeic acid	10.553	2.3629		0.399 ± 0.04
	Chlorogenic acid	9.45	16.9286		12.224 ± 0.83
	Kaempferol	4.597	0.0637	360	0.084 ± 0.14
	Quercetin	3.422	0.3127		0.054 ± 0.05
	Myricetin	2.645	26.8044		0.166 ± 0.11
ZZEE	Benzoic acid			280	ND
	Gallic acid	7.157	9.4886		0.397 ± 0.04
	Sinapic acid	11.771	3.1307		0.936 ± 0.05
	Caffeic acid	10.329	1.8612		0.14 ± 0.003
	Chlorogenic acid	9.418	16.6717		3.404 ± 0.76
	Kaempferol	4.605	0.4576	360	0.054 ± 0.005
	Quercetin	3.444	0.7825		0.042 ± 0.006
	Myricetin	2.993	39.3486		0.367 ± 0.04

Abbreviation: ZZAE—*Z. zerumbet* aqueous extract, ZAEE—*Z. zerumbet* ethanolic extract, ND-not detected, mg/g-milligram per gram.

**Table 3:** Standard flavonoid and phenolic compounds determined by the HPLC methods.

Phenolic and Flavonoid Compounds	Retention Time	Wavelength (nm)
Benzoic acid	12.97	280
Gallic acid	7.03	
Sinapic acid	11.58	
Caffeic acid	10.36	
Chlorogenic acid	9.37	
Kaempferol	4.46	360
Quercetin	3.51	
Myricetin	2.84	

**Table 4:** Antibiotic susceptibility testing of the bacterial isolates with and without the bitter ginger aqueous extracts.

Bacterial Strains	Zone of Inhibition (mm) Means ± SD					
	Without Bitter Ginger Extracts			With Bitter Ginger Aqueous Extracts		
	CIP	TE	C	CIP	TE	C
*Lactobacillus acidophilus*	18 ± 0.51 (R)	20 ± 0.48 (R)	14 ± 0.56 (R)	21 ± 0.51 (S)	24 ± 0.51 (S)	18 ± 0.48 (R)
*Streptococcus mutans*	18 ± 0.51 (R)	21 ± 0.48 (R)	16 ± 0.56 (R)	21 ± 0.51 (S)	24 ± 0.51 (S)	20 ± 0.46 (R)
*Enterococcus faecalis*	17 ± 0.51 (R)	19 ± 0.48 (R)	14 ± 0.56 (R)	19 ± 0.46 (R)	23 ± 0.5 (S)	17 ± 0.47 (R)
*Staphylococcus aureus*	15 ± 0.51 (R)	20 ± 0.48 (R)	18 ± 0.56 (R)	17 ± 0.46 (R)	24 ± 0.51 (S)	22 ± 0.50 (S)

CIP: ciprofloxacin. TE: tetracycline. C: chloramphenicol. S: sensitive. R: resistance.

**Table 5:** Antibiotic susceptibility testing of the bacterial isolates with and without the bitter ginger ethanolic extracts.

Bacterial Strains	Zone of Inhibition (mm) Means ± SD					
	Without Bitter Ginger Extracts			With Bitter Ginger Ethanolic Extracts		
	CIP	TE	C	CIP	TE	C
*Lactobacillus acidophilus*	18 ± 0.51 (R)	20 ± 0.48 (R)	14 ± 0.56 (R)	22.1 ± 0.76 (S)	25.5 ± 0.5 (S)	20.3 ± 0.57 (R)
*Streptococcus mutans*	18 ± 0.51 (R)	21 ± 0.48 (R)	16 ± 0.56 (R)	23 ± 0.5 (S)	25.3 ± 0.28 (S)	22.8 ± 0.76 (S)
*Enterococcus faecalis*	17 ± 0.51 (R)	19 ± 0.48 (R)	14 ± 0.56 (R)	21 ± 0.5 (S)	24 ± 0.5 (S)	20.5 ± 0.5 (R)
*Staphylococcus aureus*	15 ± 0.51 (R)	20 ± 0.48 (R)	18 ± 0.56 (R)	20 ± 0.5 (S)	25.8 ± 0.76 (S)	22.5 ± 0.5 (S)

CIP: ciprofloxacin. TE: tetracycline. C: chloramphenicol. S: sensitive. R: resistance.

## Data Availability

Not applicable.

## Sample Availability

Samples of the compounds are not available from the authors.
